# Supplementary material for: Short‐Term Bienenstock‐Cooper‐Munro Learning in Optoelectrically‐Driven Flexible Halide Perovskite Single Crystal Memristors
Source: Small Methods. 2025 Aug 12;9(9):e00203. doi: 10.1002/smtd.202500203 (PMC12464806; doi:10.1002/smtd.202500203)
Supplement: Supplementary file 1 — Supporting Information [file SMTD-9-e00203-s001.pdf]

small
methods

Supporting Information

for *Small Methods*, DOI 10.1002/smtd.202500203

Short-Term Bienenstock-Cooper-Munro Learning in Optoelectrically-Driven Flexible Halide Perovskite Single Crystal Memristors

Ivan Matchenya, Anton Khanas, Roman Podgornyi, Daniil Shirkin, Alexey Ekgardt, Nikita Sizykh, Sergey Anoshkin, Dmitry V. Krasnikov, Alexei Yulin, Alexey Zhukov, Albert G. Nasibulin, Ivan G. Scheblykin, Anatoly Pushkarev, Andrei Zenkevich*, Juan Bisquert* and Alexandr Marunchenko**

Supplementary for: Short-Term Bienenstock-Cooper-Munro Learning in Optoelectrically-Driven Flexible Halide Perovskite Single Crystal Memristors

Ivan Matchenya, [1,2] *Anton Khanas,* [3] *Roman Podgornyi,* [2] *Daniil Shirkin,* [2] *Alexey Ekgardt,* [2] *Nikita Sizykh,* [3] *Sergey Anoshkin,* [2] *Dmitry V. Krasnikov,* [1] *Alexei Yulin,* [2] *Alexey Zhukov,* [4] *Albert G. Nasibulin,* [1] *Ivan G. Scheblykin,* [5] *Anatoly Pushkarev,* [1,*] *Andrei Zenkevich,* [3,*] *Juan Bisquert,* [6,*] *Alexandr Marunchenko,* [2,5,*]

[1] Skolkovo Institute of Science and Technology, 30/1 Bolshoy Boulevard, 121205 Moscow, Russian Federation
[2] ITMO University, School of Physics and Engineering, St. Petersburg, 197101, Russian Federation
[3] Moscow Institute of Physics and Technology (National research university), Institutskiy per. 9, Dolgoprudny, Moscow Region, 141701, Russian Federation
[4] International Laboratory of Quantum Optoelectronics, HSE University, Soyuz Pechatnikov str. 16, St.Petersburg, 190008, Russian Federation
[5] Chemical Physics and NanoLund, Lund University, P.O. Box 124, 22100 Lund, Sweden
[6] Instituto de Tecnología Química (Universitat Politècnica de València-Agencia Estatal Consejo Superior de Investigaciones Científicas), 46022 València, Spain
Andrey Zenkevich
Email Address: zenkevich.av@mipt.ru
A.P. Pushkarev
Email Address: an.pushkarev@skoltech.ru
Juan Bisquert
Email Address: bisquert@uji.es
A.A. Marunchenko
Email Address: a.marunchenko@metalab.ifmo.ru

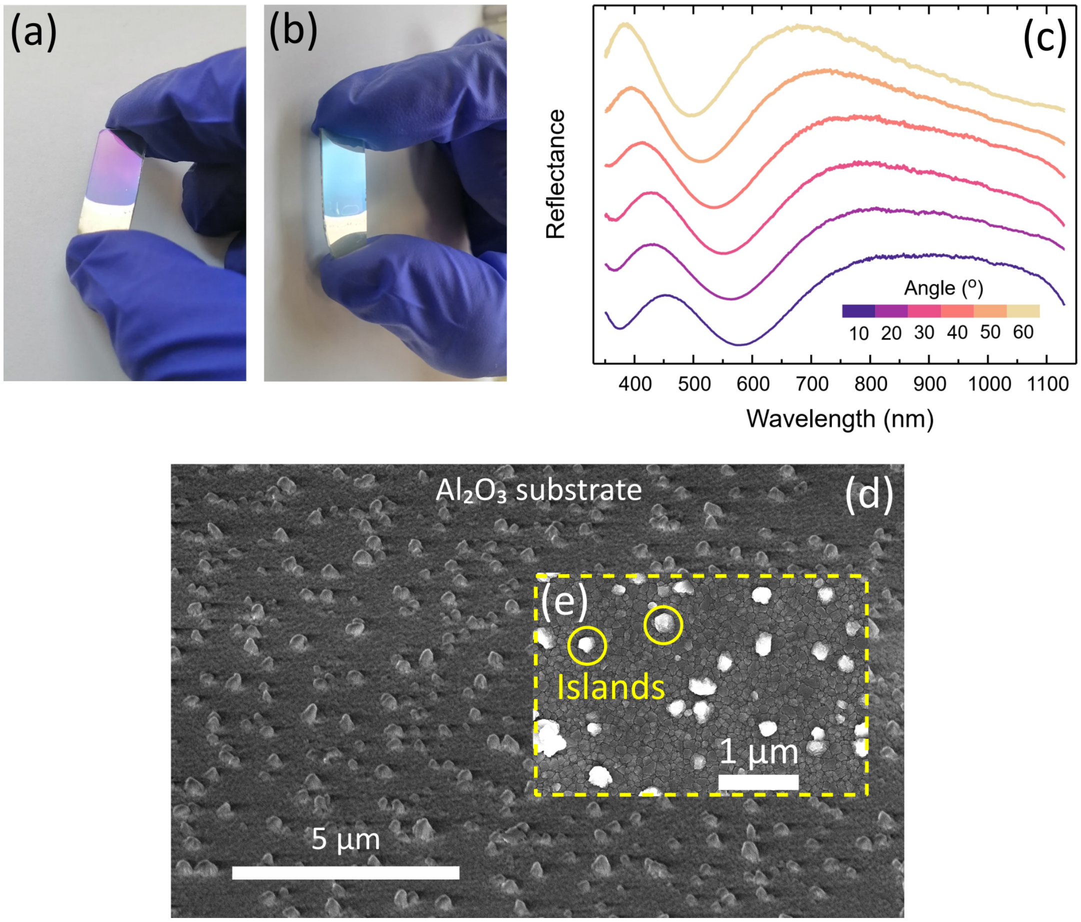

Figure S1: **Characterization of nanostructured Al_2O_3 substrates. (a-b)** Images of the nanostructured Al_2O_3 substrate with island-like morphology. **(c)** Reflectance spectra of the Al_2O_3 substrate at different angles. **(d)** SEM and **(e)** HRSEM images of the Al_2O_3 substrate highlighting formation of islands.

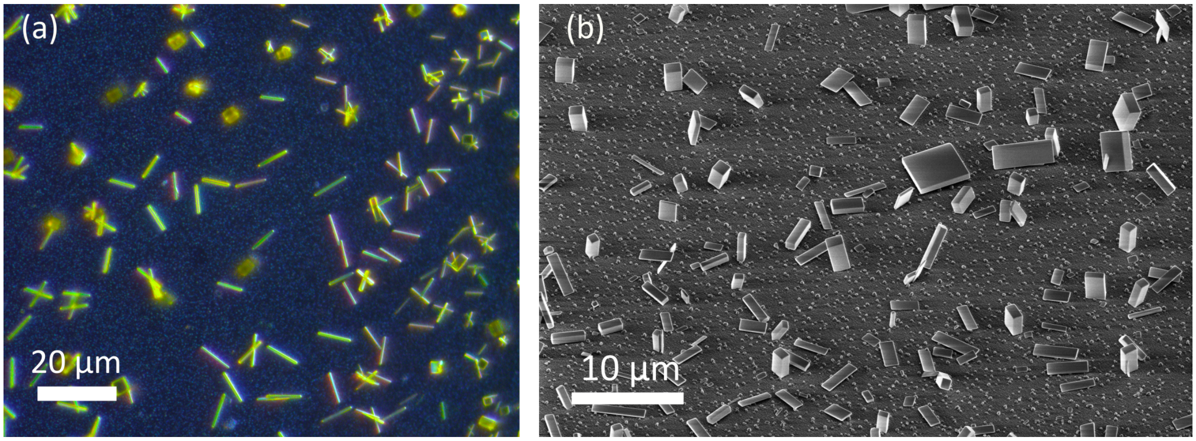

Figure S2: **Synthesis of the $CsPbBr_3$ microcrystals. (a-b)** Dark-field and SEM images of microcrystals grown on the Al_2O_3 substrate

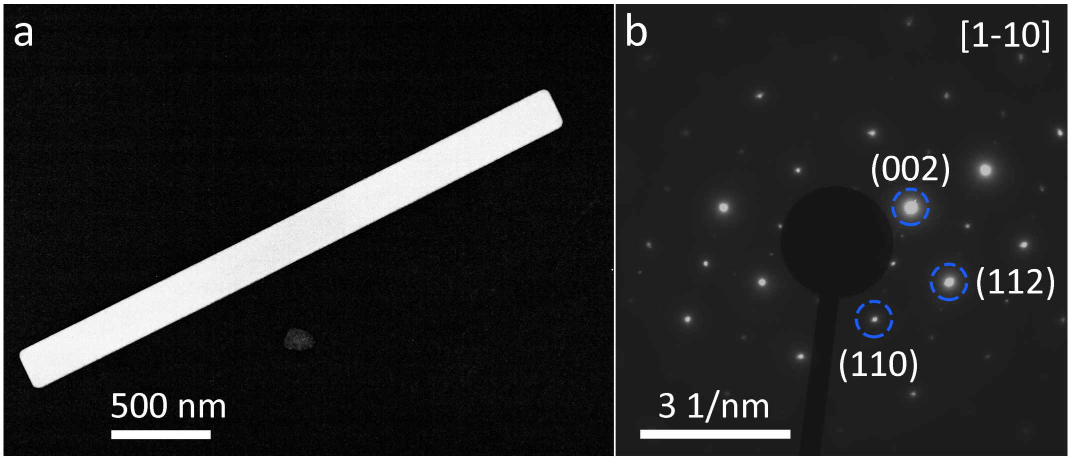

Figure S3: **Establishing the monocrystallinity of a single perovskite microcrystal.** **(a)** Low-resolution HAADF-STEM image of a single microwire. **(b)** SAED image of the entire microwire revealing sharp diffraction spots assigned to planes of perovskite lattice and confirming the microwire monocrystallinity

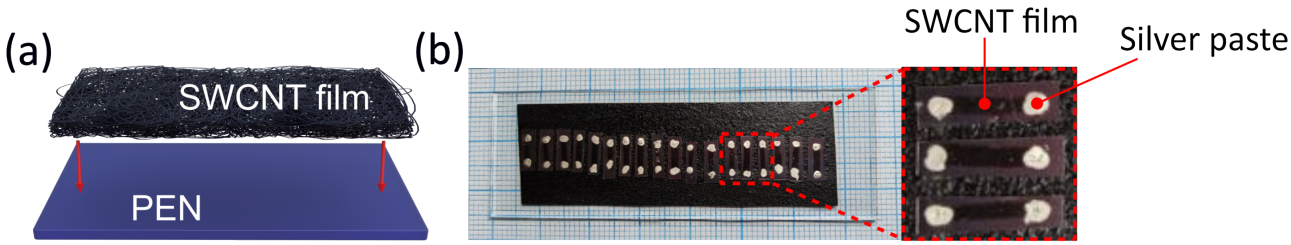

Figure S4: **(a)** Transfer of SWCNT film to the PEN substrate, **(b)** prepared PEN substrate with transferred SWCNT film and droplets of silver paste for laser ablation

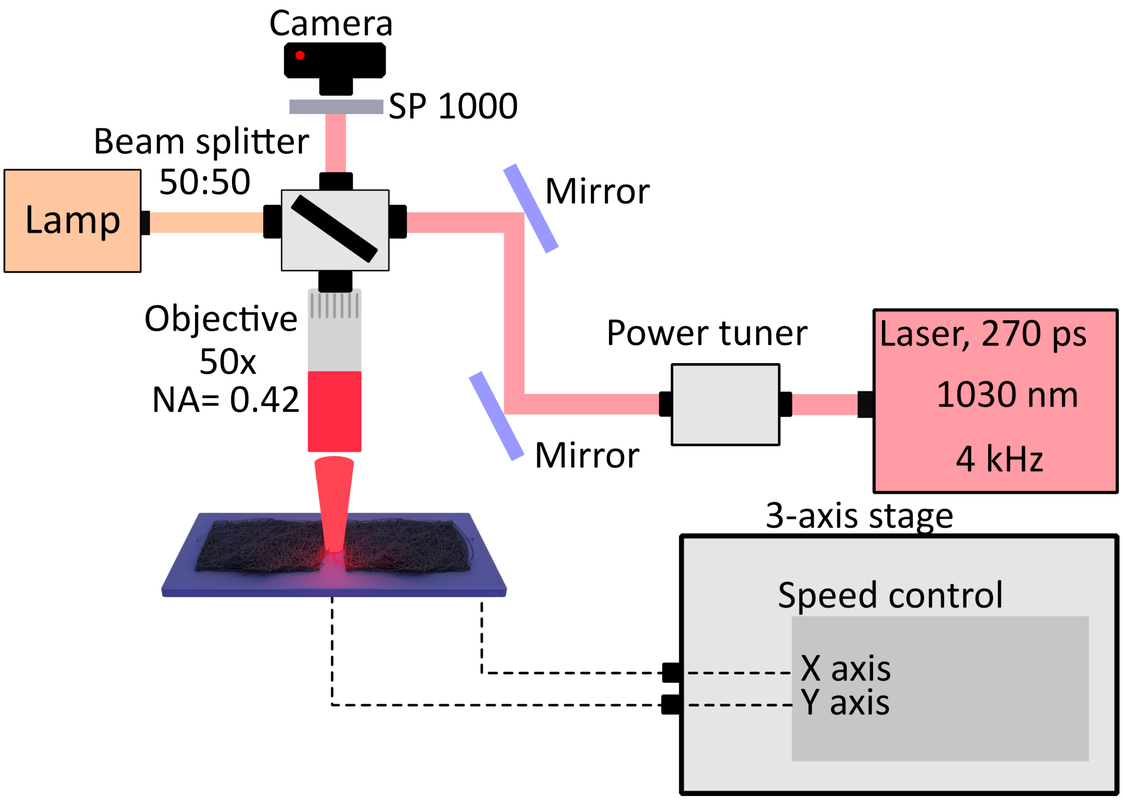

Figure S5: **Optical scheme for laser ablation.** The velocity and acceleration of the Standa 3-axis stage were chosen in such a way that there were no short-circuits between the SWCNT electrodes.

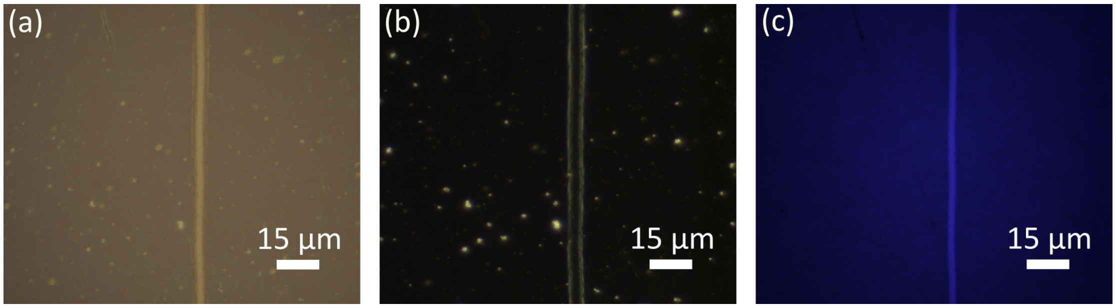

Figure S6: **SWCNT electrodes.** Bright-field **(a)**, dark-field **(b)** and fluorescent dark-field image **(c)** of the SWCNT electrodes after laser ablation

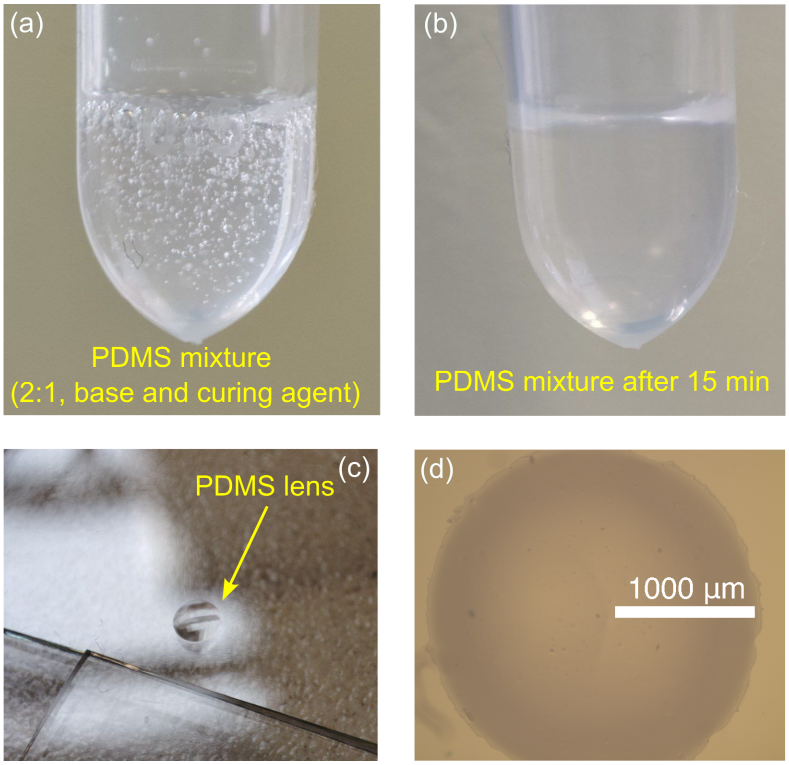

Figure S7: **Process of making a PDMS lens for the dry transfer process. (a)** Photo of mixture of the base viscous polymer component and a curing agent (2:1 mass ratio) after stirring, **(b)** photo of mixture after 15 min degassing, **(c)** photo and **(d)** bright-field image of PDMS drop on glass substrate

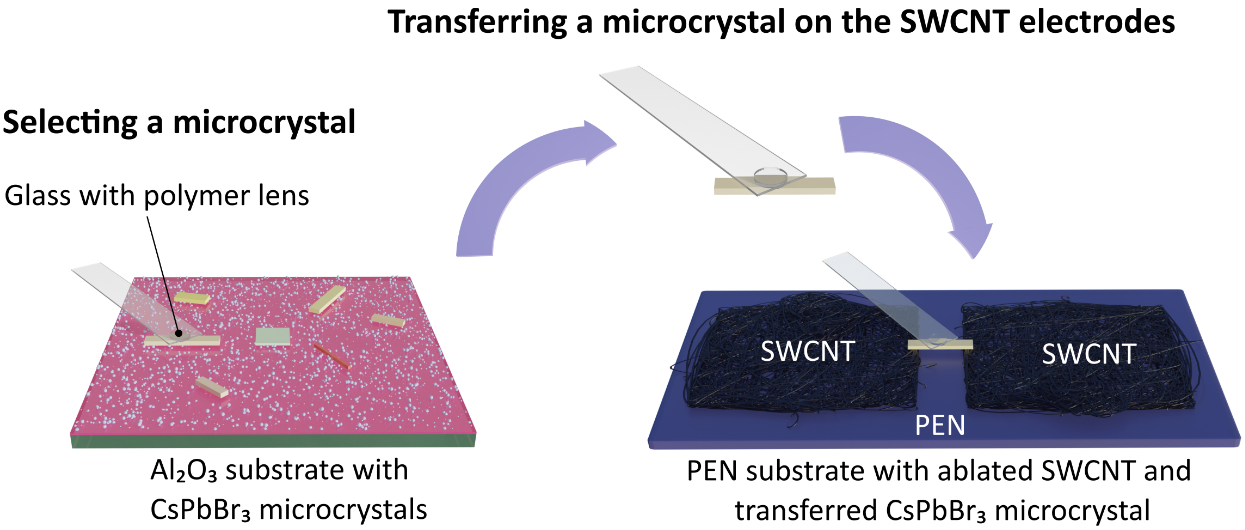

Figure S8: **Schematic illustration of the transfer process.**

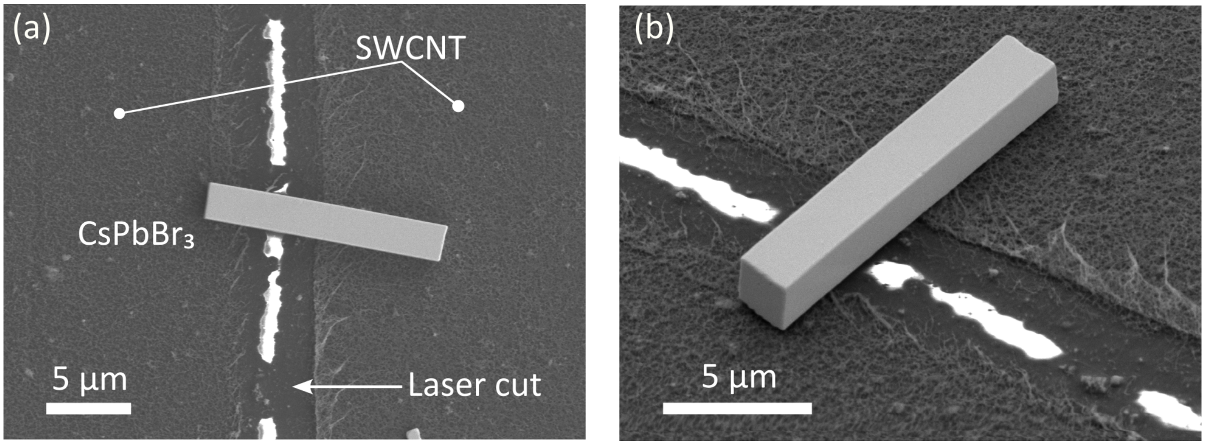

Figure S9: **Image of the actual device**. **(a)** Top-view and tilted-angle **(b)** SEM-image of $CsPbBr_3$ microscrystal memristor bridging SWCNT electrodes. Image from **(b)** is magnified Figure 2l from the main text. White lines in the cut area appeared due to the accumulation of charge on the polymer substrate.

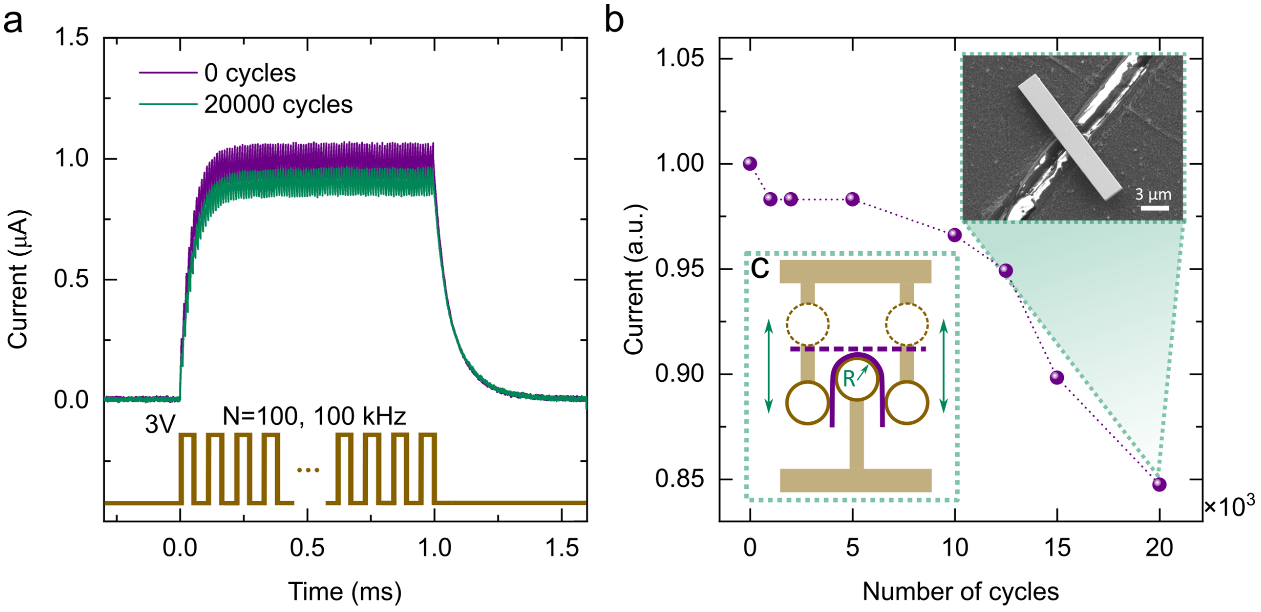

Figure S10: **Contact stability under optoelectronic stimuli combined with mechanical bending for 20000 cycles.** **(a)** Optoelectronic stimuli consist of 100 pulses of 5 μs at 3 V and the repetition rate of 100 kHz and 30 mW/cm^2 light illumination at the wavelength of 532 nm. **(b)** The normalized response of the device to this optoelectrical stimuli under mechanical bending of a tunable number of bending cycles. The device response is measured after each 5000 bending cycles. SEM image (right inset) of the device after 20 000 bending cycles shows no visible changes in the morphology of the crystal. White lines in the cut area appeared due to the accumulation of charge on the polymer substrate. **(c)** Schematic front-view illustration of cylindrical-based bending machine. Curvature radius is equal to R = 2 mm. Arrows indicate direction of movement of cylinders.

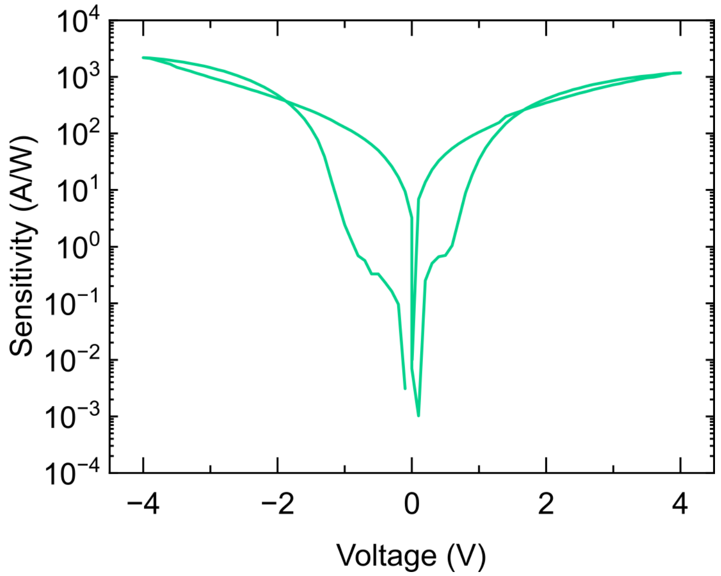

Figure S11: **Voltage-dependent sensitivity of the photodetecting microcrystal.**

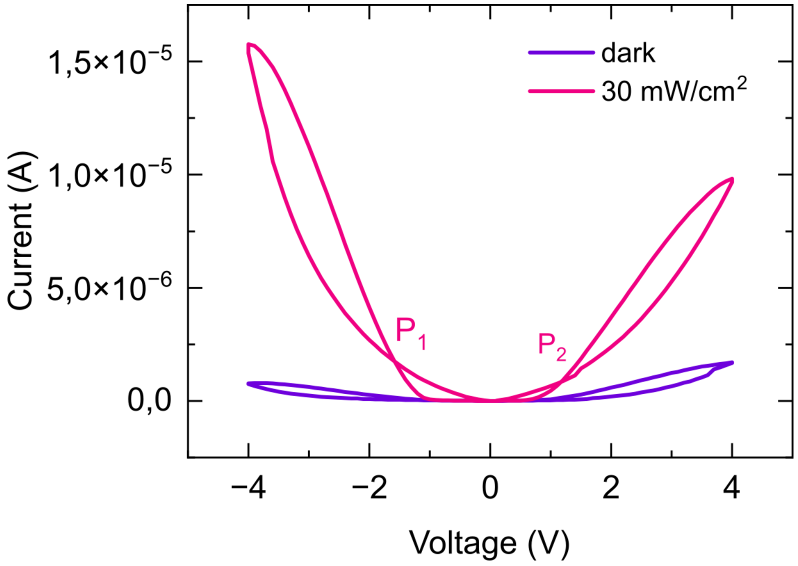

Figure S12: **Current-voltage characteristics in linear scale.**

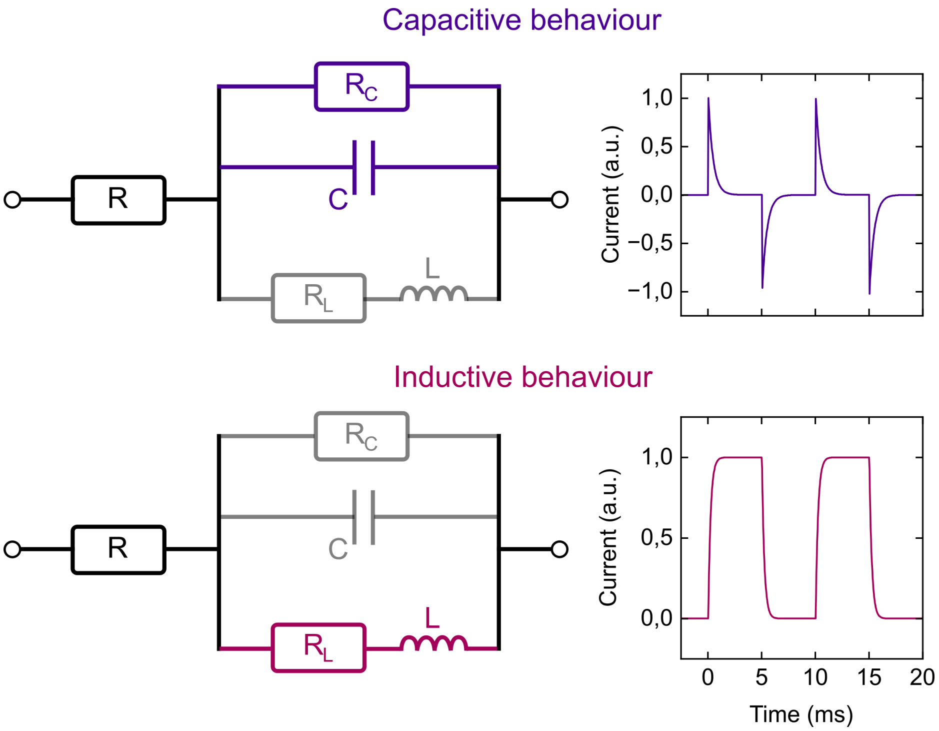

Figure S13: **Equivalent circuit model with capacitive and inductive branches and corresponding transient responses.**

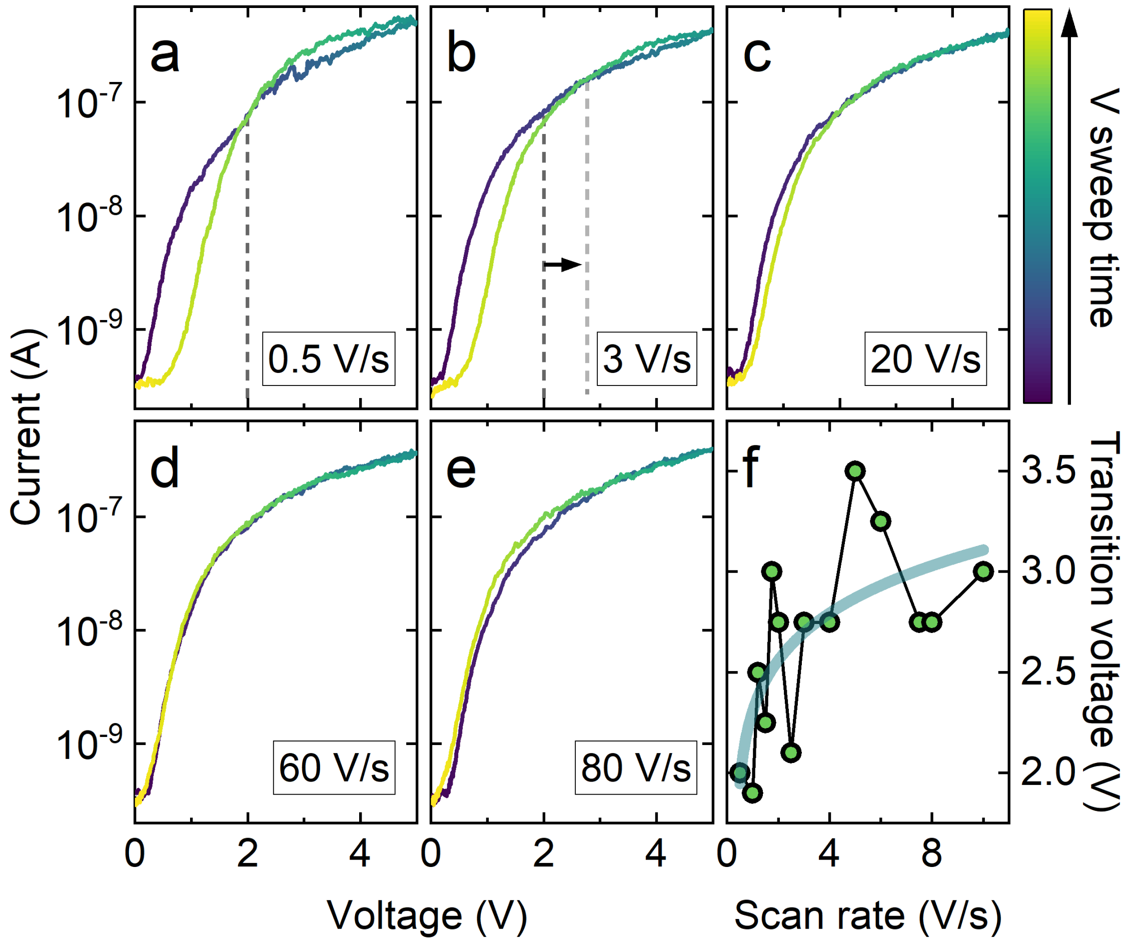

Figure S14: **Scan-rate analysis of the I-V curve. (a-e)** I-V curves at different scan-rates. **(f)** Dependence of the transition voltage (P_2 point from Figure 2l of the main text and Figure S12) on the scan rate

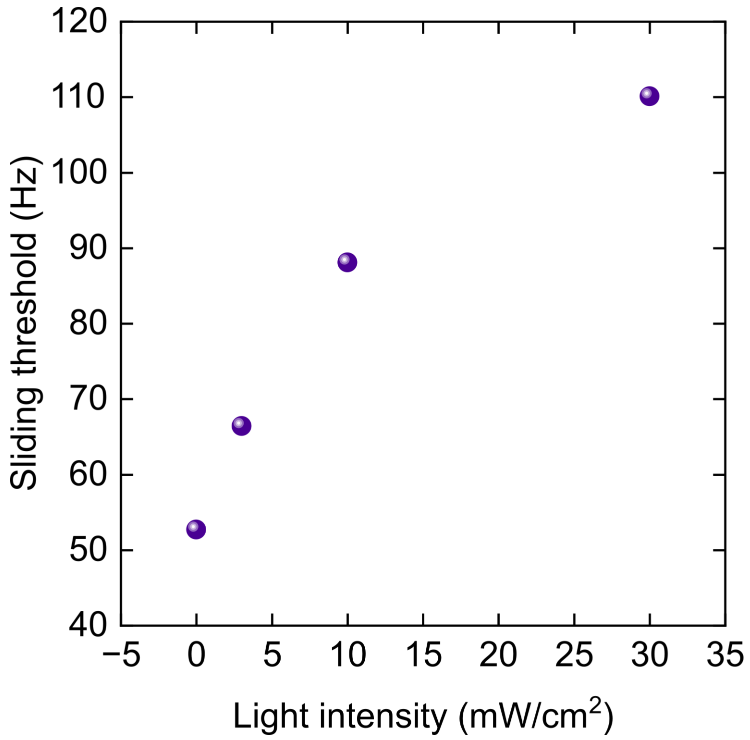

Figure S15: **The illumination intensity-dependent sliding threshold obtained from BCM experiment.**

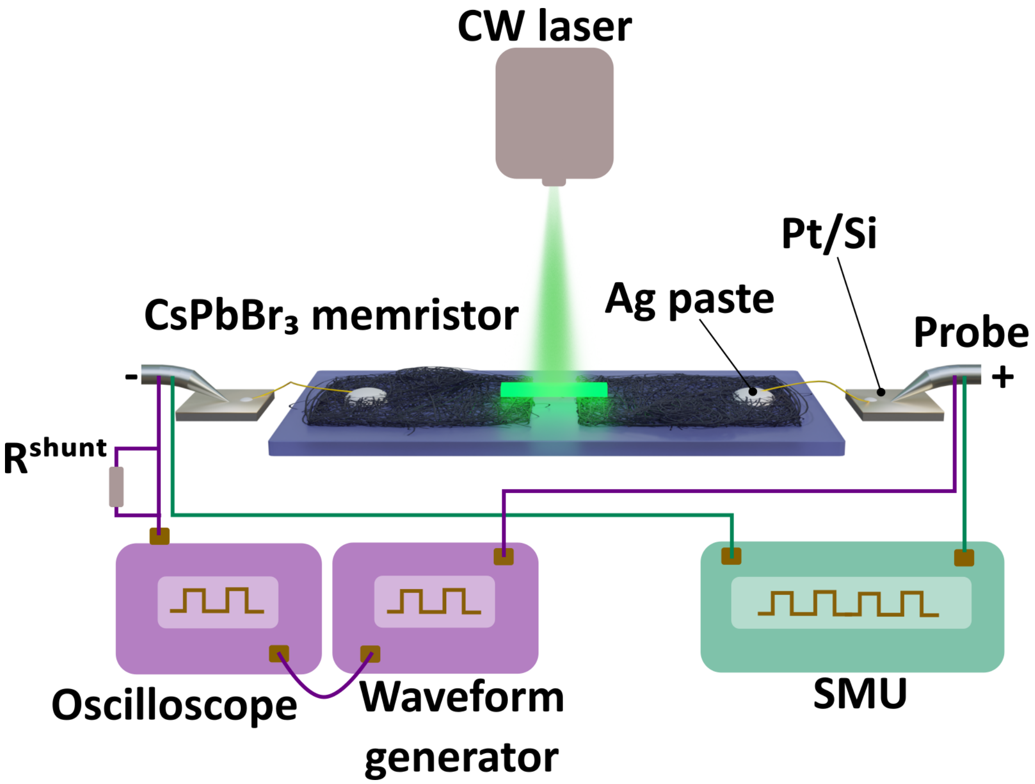

Figure S16: **Schematic setup for the optoelectrical experiments.**

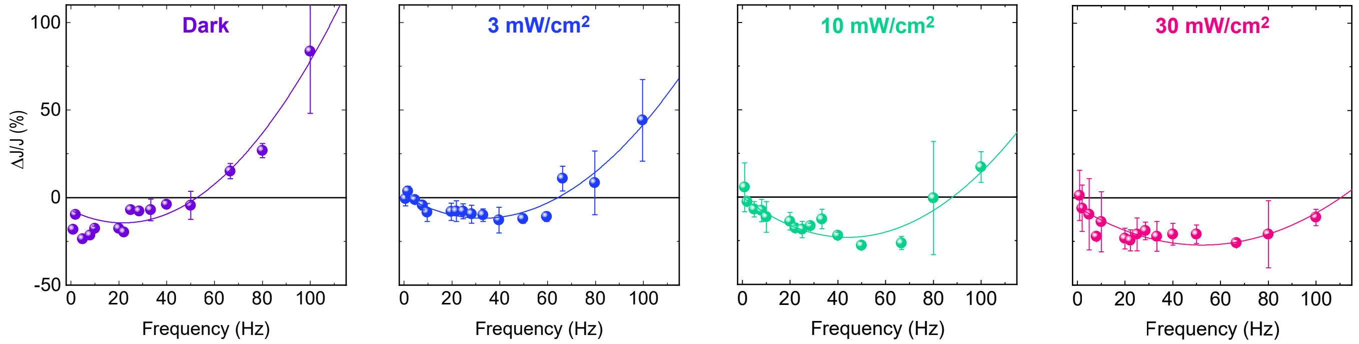

Figure S17: **Statistical BCM measurements.**

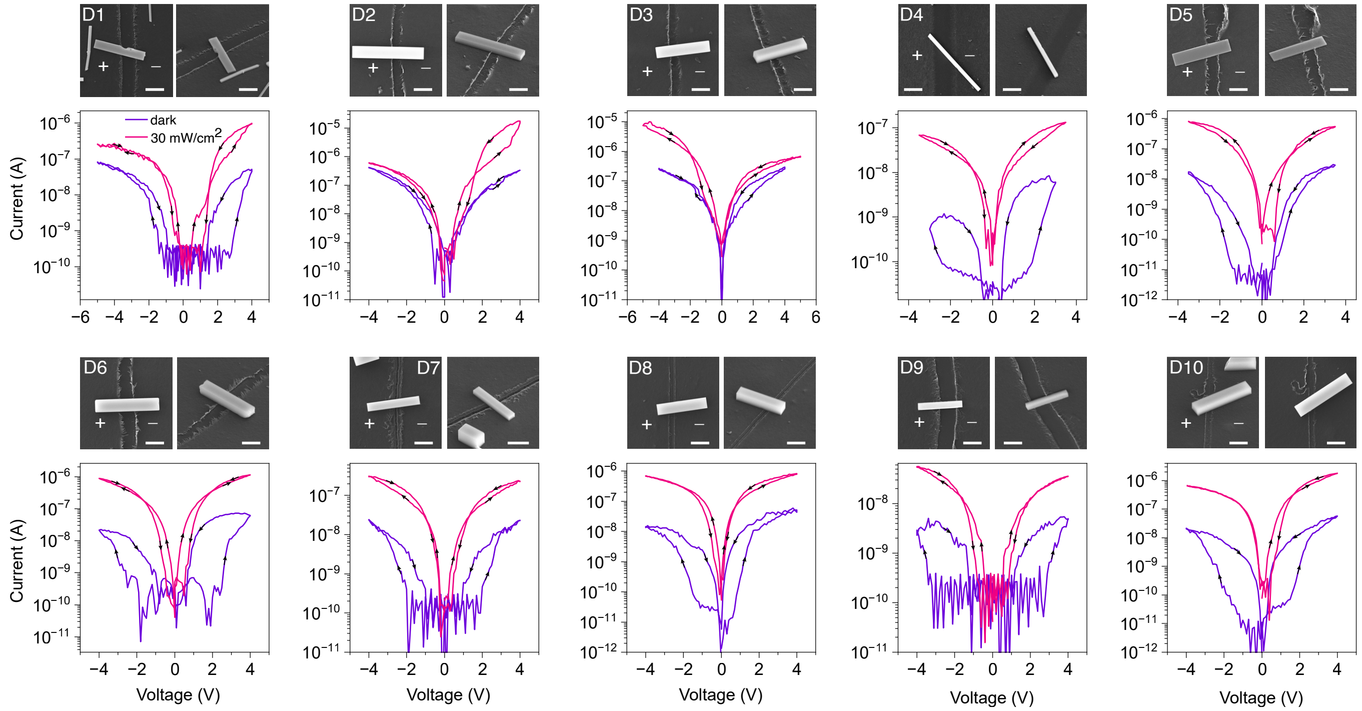

Figure S18: **Top-view and tilted-angle SEM images of ten additional devices, together with their corresponding I–V curves measured in the dark (purple) and under 30 mW/cm^2 CW laser illumination (pink).** Arrows indicate the voltage-sweep directions. All devices exhibit inductive behavior in the dark, transitioning to mixed inductive–capacitive behavior under illumination. Scale bar is 5 μm in all panels.
